# Concomitant Acute Ischemic Stroke and Upper Extremity Arterial Occlusion: Feasibility of Mechanical Thrombectomy of the Upper Limb Using Neurointerventional Devices and Techniques

**DOI:** 10.3390/jcm10143189

**Published:** 2021-07-20

**Authors:** Dominik F. Vollherbst, Christian Ulfert, Volker Maus, Timan Boujan, Hans Henkes, Martin Bendszus, Markus A. Möhlenbruch

**Affiliations:** 1 Department of Neuroradiology, Heidelberg University Hospital, 69120 Heidelberg, Germany; dominik.vollherbst@med.uni-heidelberg.de (D.F.V.); christian.ulfert@med.uni-heidelberg.de (C.U.); martin.bendszus@med.uni-heidelberg.de (M.B.); 2 Department of Radiology, Neuroradiology and Nuclear Medicine Ruhr-University Bochum, Knappschaftskrankenhaus, Bochum-Langendreer, 44892 Bochum, Germany; volker.maus@kk-bochum.de; 3 Department of Radiology, Hospital Ludwigshafen, 67063 Ludwigshafen, Germany; boujant@klilu.de; 4 Neuroradiological Clinic, Klinikum Stuttgart, 70174 Stuttgart, Germany; hhhenkes@aol.com

**Keywords:** ischemic stroke, large vessel occlusion, upper limb ischemia, upper extremity arterial occlusion, mechanical thrombectomy

## Abstract

Background: Concomitant acute ischemic stroke (AIS) caused by large vessel occlusion (LVO), and acute upper extremity arterial occlusion causing upper limb ischemia (ULI) is a rarely observed coincidence. The first-line treatment for AIS is mechanical thrombectomy (MT), with or without additional intravenous thrombolysis, while there are different pharmacological, surgical and endovascular treatment options for an acute occlusion of the UL arteries. Here, we describe the practicability, efficacy and safety of neurointerventional devices and techniques for MT of upper extremity arterial occlusions. Materials and Methods: A retrospective analysis of prospectively collected patient databases from four neurovascular centers was performed. Clinical and imaging data, as well as procedural parameters, were assessed. Results: Seven out of 6138 patients (incidence: 0.11%) presenting with an AIS due to the occlusion of craniocervical arteries requiring MT and a concomitant occlusion of the brachial (4/7), axillary (2/7), or ulnar (1/7) artery causing acute ULI were identified. Craniocervical MT was technically successful in all cases. Subsequent MT of the upper limb was performed using neurointerventional thrombectomy techniques, most frequently stent retriever thrombectomy (in 4/7 cases) and direct aspiration (in 7/7 cases). MT achieved successful recanalization in 6/7 cases, and the UL completely recovered in all six cases. In one case, recanalization was not successful, and the patient still had a marginally threatened extremity after the procedure, which improved after pharmacological therapy. Conclusion: In the rare case of AIS requiring MT and concomitant acute upper extremity arterial occlusion, MT of the UL arteries using neurointerventional devices and techniques is practical, effective, and safe.

## 1. Introduction

Together with intravenous thrombolysis, endovascular mechanical thrombectomy (MT) has become the first-line treatment for acute ischemic stroke (AIS) caused by large vessel occlusion (LVO) of the brain-supplying arteries [1]. Numerous studies have investigated the indications for MT and different MT techniques, which are now established to treat LVO [2,3,4]. Another site which LVOs can occur is the upper extremity. There are several different treatment options for an acute upper extremity arterial occlusion causing acute upper limb ischemia, which include pharmacological, surgical and endovascular approaches [5,6,7]. A rarely reported coincidence is an LVO of the craniocervical arteries causing an AIS, and a concomitant acute occlusion of the upper extremity arteries causing acute upper limb ischemia [8,9]. The ischemic tolerance of the brain is much lower than that of the extremities, which is why the LVO causing AIS should always be targeted first. After completion of the MT of the craniocervical LVO, it may be necessary to transfer the patient to another operation room, another medical department, or even to another hospital for the treatment of the upper extremity arterial occlusion. This transfer can be very time-consuming, and can substantially impair both the outcome of the patient’s upper limb and also the neurological outcome of the patient. A reasonable treatment option would appear to be the treatment of a concomitant upper extremity arterial occlusion in the same treatment session as applying neurointerventional MT techniques using the associated devices. However, this approach has not yet been the focus of research. This study aimed to assess the practicability and efficacy of neurointerventional devices and techniques for mechanical thrombectomy of acute upper extremity arterial occlusions.

## 2. Materials and Methods

Institutional review board approval was received, according to the guidelines of the local ethics committee. The need for individual patient consent was waived by each site due to the retrospective nature of this study. A retrospective analysis of prospectively maintained stroke databases for four high-volume neurointerventional centers was performed to identify all patients who presented with a concomitant MT-treated AIS, and an upper extremity arterial occlusion treated by endovascular means in the same treatment session. Patients with chronic occlusions of the upper extremity arteries, either pre-known or suspected by computed tomography angiography (CTA), were not included in this analysis.

Patient demographics, cardiovascular risk factors, neurological presentation (pre-morbid modified Rankin Scale (mRS) and National Institutes of Health Stroke Score (NIHSS)), and symptoms of the upper extremity arterial occlusion were assessed. The location of the craniocervical and the upper extremity arterial occlusions were recorded, along with the Alberta stroke program early CT score (ASPECTS) [10]. For the assessment of the pre- and post-interventional perfusion of the affected vessels, the modified Thrombolysis in Cerebral Infarction (mTICI) scale was used for the cerebral vessels, and the Thrombolysis in Limb Infarction (TILI) scale was used for the upper extremity vessels [11,12]. The TILI scale was defined as follows: TILI 0: no reperfusion, TILI 1: No filling of distal vessels, TILI 2a: reperfusion of >1/3 of the vascular territory of the initially occluded vessel, TILI 2b: residual perfusion deficits in ≤1/3 of the vascular territory of the initially occluded vessel, TILI 3: complete reperfusion with filling of all distal vessels. Technical success was defined as the successful completion of the procedure and mTICI ≥ 2b for the craniocervical occlusion and TILI ≥ 2b for the upper extremity arterial occlusion. The number of MT maneuvers, the MT techniques, the MT devices and the procedure times were recorded for the craniocervical and the upper extremity occlusions, respectively. Post-interventional assessments included the etiology of the stroke (according to the TOAST classification [13]), the clinical status of the extremity on discharge (according to the Rutherford classification [14]), and the neurological outcome of the patients (NIHSS at discharge and mRS at discharge and 3 months).

## 3. Results

A total of 6138 patients diagnosed with an AIS due to an LVO were treated by MT in four neurovascular centers between January 2016 and February 2021. Of this population, seven patients presented with a concomitant upper extremity arterial occlusion, which was also treated by endovascular means in the same treatment session, resulting in an estimated incidence of 0.11%. Patients who presented with this coincidence and in whom the upper extremity arterial occlusion was not treated endovascularly (e.g., surgical treatment or no treatment) could not be identified.

The baseline characteristics are summarized in Table 1. Mean age was 76.4 ± 10.2 years, with a female–male ratio of 4:3. Mean baseline NIHSS was 16 ± 6 (range, 9–24), and the mean baseline ASPECTS was 8 ± 2 (range, 6–10). Arterial hypertension (6/7) and atrial fibrillation (5/7) were the most frequently present cardiovascular risk factors. A large thrombus burden, defined as thrombus, which could not be completely removed with one productive MT maneuver, was observed in 5 of the 7 patients. These patients presented either with more than one LVO (patients #2 and #3), with an occlusion of the common carotid artery (CCA) or with a carotid T occlusion. The most frequent location of the upper extremity arterial occlusion was the brachial artery (4/7), followed by the axillary (2/7) and the ulnar artery (1/7). In 5/7 patients, cardioembolism was the most likely etiology of the stroke.

The procedural characteristics of the craniocervical occlusions are summarized in Table 2. Femoral access was used for all interventions. The MT of the craniocervical occlusion was technically successful in all cases. For the craniocervical occlusions, 4.1 ± 3.5 MT maneuvers were required for reperfusion, ranging from one to nine maneuvers. Especially for the patients with large thrombus burden, multiple maneuvers had to be performed (*n* = 9 in patient #1, *n* = 3 in patient #2, *n* = 9 in patient #3, and *n* = 1 in patients #6 and #7, respectively). In one case (patient #2), PTA and stenting of a high-grade stenosis of the proximal internal carotid artery was required. During craniocervical MT, one complication occurred: a dissection of the cervical vertebral artery (V2-segment), which was treated pharmacologically by the administration of heparin and aspirin. Due to the only minor hemodynamic effects and good flow via the contralateral vertebral artery, no interventional treatment was necessary.

**Figure 1:**
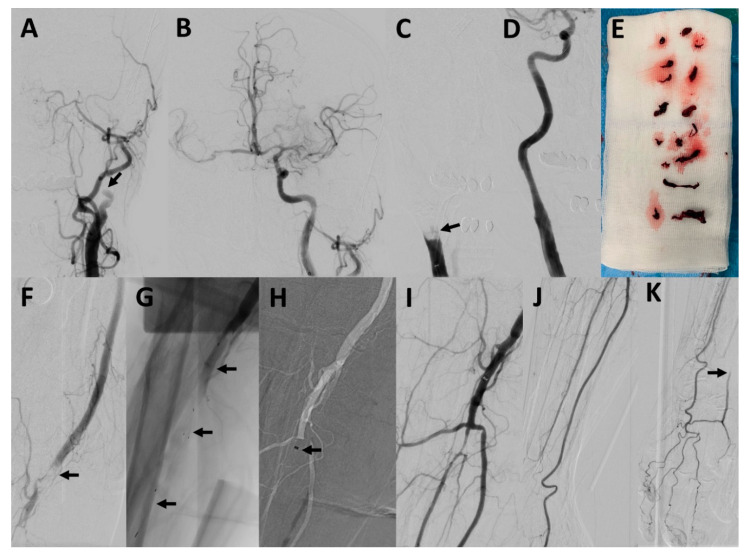
Mechanical thrombectomy of concomitant occlusions of the internal carotid artery, common carotid artery and brachial artery. Patient #2 presented with a left-sided occlusion of the internal carotid artery ((**A**), arrow), a right-sided occlusion of the common carotid artery ((**C**), arrow) and an occlusion of the right brachial artery (**F**, arrow). An occlusion of the V2-segment of the left vertebral artery was also present (not shown) but was not recanalized. After successful recanalization of the left internal carotid artery using direct aspiration (**B**); with only poor collateralization of the territory of the right internal carotid artery) and of the right common carotid artery using aspiration, balloon dilatation and stenting (**D**), harboring a lot of thrombus material (**E**), the right upper limb occlusion was targeted. The brachial artery could be recanalized using one stent retriever maneuver (Neva-T stent retriever marked with arrows in (**G**) and 3 aspiration maneuvers (arrow pointing at the tip of a 6F Sofia aspiration catheter in (**H**)). Thrombectomy of the brachial artery was technically successful (**I**), also harboring several large thrombi (**E**). The radial artery could not be completely recanalized, but was filled retrogradely (**J**,**K**).

**Figure 2:**
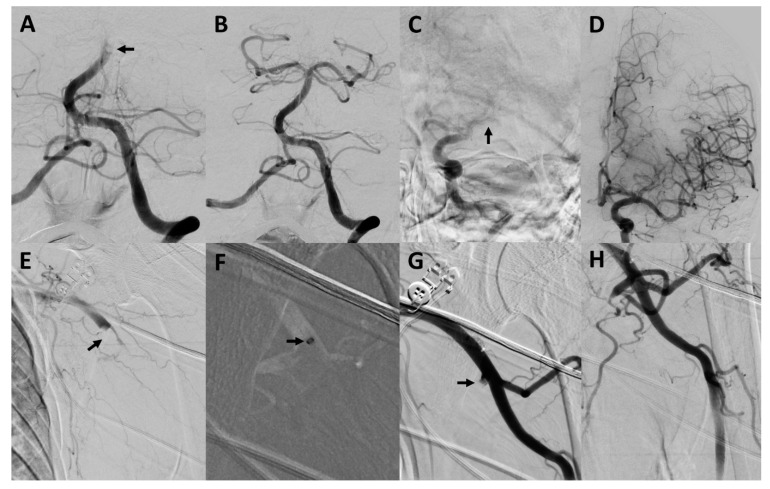
Mechanical thrombectomy of concomitant occlusions of the basilar artery, middle cerebral artery and axillary artery. Patient #3 presented with an occlusion of the distal basilar artery ((**A**), arrow), a left-sided occlusion of the M1-segment of the middle cerebral artery ((**C**), arrow) and an occlusion of the left-sided distal axillary artery ((**E**), arrow). After successful recanalization of the basilar (**B**) and middle cerebral artery (**D**), recanalization of the axillary artery was performed. In total, 4 direct aspiration maneuvers were performed for recanalization (arrow in (**F**) pointing at the tip of a 9F Merci catheter), including an aspiration maneuver for recanalization of the subscapular artery using a 6F Sofia aspiration catheter (arrow in (**G**)). An angiogram of the recanalized axillary artery is presented in (**H**).

**Figure 3:**
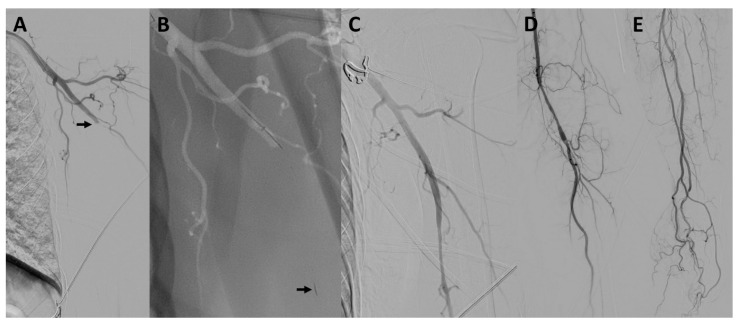
Mechanical thrombectomy of concomitant occlusions of the left carotid T and the axillary artery. Patient #6 presented with an occlusion of the carotid T, which could be successfully recanalized with one thrombectomy maneuver (not shown). Digital subtraction angiography showed a concomitant occlusion of the left axillary artery (arrow in (**A**)). A microwire/microcatheter was navigated into the occluded vessel and an Embo Trap II 5 × 33 mm stent retriever was deployed within the occlusion (arrow pointing at the distal tip of the stent retriever in (**B**)). The occlusion was successfully recanalized with one stent retriever maneuver without any residual occlusion (**C**–**E**).

The procedural characteristics of the upper extremity arterial occlusions are also summarized in Table 2. Three example cases are illustrated in Figure 1, Figure 2 and Figure 3. The procedure time for the upper extremity arterial occlusions was 38.9 ± 22.4 min. The number of MT maneuvers performed was 2.9 ± 1.4, ranging from 1 to 4 maneuvers. MT using a stent retriever was performed in 4/7 cases, while MT using direct aspiration was performed at least once in every case. In one case, after several unsuccessful MT maneuvers with conventional techniques, a double stent retriever thrombectomy, with two stent retrievers positioned parallel in the occluded vessel, was successfully performed. In a further case, after several unsuccessful MT maneuvers, the distal brachial artery could be recanalized by wire manipulation, using the tip of a 0.035-inch guidewire (Radifocus Glidewire Advantage; Terumo, Leuven, Belgium). This maneuver resulted in a transient perforation without any flow disruption or clinically visible or palpable hematoma. No further complications were observed. MT of the upper extremity arterial occlusion was technically successful in 6/7 cases (85.7%). Of the successful cases, complete reperfusion with filling of all distal vessels (TILI 3) was achieved in only one patient, and there were residual perfusion deficits (TILI 2b) in the remaining five cases. The case in which the recanalization was not successful was an occlusion of the ulnar artery, which persisted after a direct aspiration thrombectomy attempt. A pharmacological treatment with intravenous prostaglandin E1 for 4 days was started immediately after the procedure.

The outcome parameters are summarized in Table 3. One patient developed an intracerebral hemorrhage into the infarcted brain tissue after the procedure. A favorable neurological outcome (mRS ≤ 2 at 3 months) was achieved in 2/7 patients. In all cases with technically successful MT of the upper extremity arterial occlusion, the extremity was viable after the intervention. Only in the case in which the MT was not successful was the extremity was still marginally threatened postinterventionally. The pharmacological treatment resulted in an improvement in the extremity and rendered any further interventional therapy unnecessary.

The post-procedural medical antithrombotic management consisted of 100 mg of aspirin daily (lifelong) for all patients and additionally 75 mg of clopidogrel daily (for 30 days) for patient #2 because of the carotid artery stenting.

## 4. Discussion

Concomitant AIS caused by an LVO and an acute upper extremity arterial occlusion causing acute upper limb ischemia is a rarely observed coincidence [8,9]. Treating the LVO causing AIS first is of the utmost importance. However, the subsequent treatment of the upper extremity occlusion can be managed differently.

This study examined data from seven patients treated in four high-volume neurovascular centers. After the completion of endovascular treatment of an AIS, MT of the upper limb arteries in the same treatment session, using similar neurointerventional devices and techniques, was technically and clinically successful in 6 out of 7 cases, with only one minor complication.

Based on the fact that 5 out of 7 patients presented with a large thrombus burden, and cardioembolic stroke was the suspected etiology in another five patients (different patient subsets), we can suspect that large cardiac thrombi might be the reason for this dual event. After detachment of the thrombus in the heart, shattering of the thrombus in the aortic arch with subsequent multiple vessel occlusions is a possible explanation for the investigated coincidence. However, the relatively low case numbers in this study do not allow any certain conclusions to be drawn regarding the etiology of the investigated coincidence.

The first-line treatment for AIS caused by an LVO is mechanical thrombectomy (MT), while there are several different pharmacological, surgical and endovascular treatment options for an acute occlusion of the upper limb arteries. A common pharmacological treatment option is the selective endovascular infusion of thrombolytic drugs, such as recombinant tissue-type plasminogen activator (rt-PA), into the occluded vessel [15]. One of the most frequently applied surgical treatments is thrombo-embolectomy, commonly using the Fogarty technique: transverse brachial arteriotomy in the fossa cubiti and removal of thromboembolic material with a balloon catheter [16]. An effective endovascular recanalization technique is rheolytic thrombectomy [17,18]. The mechanism of rheolytic thrombectomy is a high-pressure saline jet in conjunction with aspiration. All of these treatment options carry certain potential drawbacks, which limit their application in the case of a concomitant AIS. The admission of rt-PA, which is often also used systemically for the treatment of the AIS, can increase the risk of intracranial hemorrhage, and is thus contraindicated in most cases. Surgical thrombo-embolectomy usually requires the transfer of the patient to another department or to another operation room, which can be time-consuming and impair the outcome of the patient’s limb. Moreover, any additional surgical procedure after a stroke may interfere with the neurological recovery of the patient. Neurointerventionalists do not regularly perform rheolytic thrombectomy, and the devices for rheolytic thrombectomy are often not available in the neurointerventional angiography suite. However, this technique might be a reasonable alternative if MT using neurointerventional techniques fails.

In the case of concomitant craniocervical LVO and an acute upper extremity arterial occlusion, after completion of the neurointerventional procedures, the arterial access route is already established, and the devices are already unpacked or even in place for MT of the upper extremity occlusion, which makes this approach highly time- and cost-effective. According to the results of our study, the neuro-interventionalist can effectively use the devices and techniques for the upper extremities, similar to cerebral thrombectomy. More aggressive MT techniques, which bear a higher risk of hemorrhagic complications in the brain, such as wire manipulation or double stent retriever techniques, can be used earlier and more liberally in the limb because of the comparatively far lower impact of hemorrhagic complications.

Data on MT of arterial limb occlusion using neurointerventional techniques or devices are rare. One study investigating MT of acute arterial limb occlusions by applying MT using a retrievable stent, without the coincidence of a concomitant AIS, was published by Zhou et al. [11]. In this study, they report 17 patients suffering from acute occlusions of the upper extremity arteries, treated using the Solitaire AB stent (Medtronic, Minneapolis, MN, USA). The occluded arteries could be successfully recanalized in 88.2% of the cases, with a mean number of three thrombectomy maneuvers. For neurointerventional MT procedures, the Solitaire AB stent was replaced by newer devices, such as the Solitaire X stent retriever (Medtronic) or other stent retrievers of newer generations from other manufacturers. Furthermore, in the study by Zhou et al., the access devices are different from those of neurointerventional MT procedures (e.g., triaxial system, balloon-guide catheters, aspiration catheters and low-profile microcatheters).

This study has several limitations. Given the low frequency of the investigated coincidence, the case numbers are low. However, this study includes data from four high-volume neurovascular centers, and is the largest study reporting on this topic at present. Furthermore, certain bias exists as a result of the retrospective nature of this work. Another drawback is the lack of imaging follow-up (e.g., ultrasonography) and the lack of clinical mid- or long-term follow-up of the patients’ limbs.

## 5. Conclusions

Concomitant LVO causing AIS, and acute upper extremity arterial occlusion causing acute upper limb ischemia, is a rarely observed coincidence. After completing the MT of the LVO, MT of the upper limb arteries in the same treatment session, using neurointerventional devices and techniques, is practical, effective, and safe for patients presenting with this coincidence.

## Figures and Tables

**Table 1:** Baseline characteristics.

**Characteristic**	Patient No.						
	1	2	3	4	5	6	7
Age	85	71	83	81	68	60	87
Sex	Female	Female	Male	Male	Male	Female	Female
NIHSS	9	19	24	11	20	12	15
Pre-morbid mRS	0	1	1	1	0	0	3
Arterial hypertension	Yes	Yes	No	Yes	Yes	Yes	Yes
Diabetes mellitus type 2	No	No	No	No	Yes	No	Yes
Smoker	No	Yes	No	No	Yes	No	No
Previous stroke	No	No	No	Yes	No	No	No
ASPECTS	8	7	10	8	9	10	6
I.v. thrombolysis ^1^	Yes	Yes	Yes	No	No	No	No
Location of craniocervical occlusion	Right CCA	Left ICA, right CCA, right VA-V2 ^3^	BA, left MCA-M1 ^4^	BA	Right MCA-M1	Left carotid T	Right carotid T
Location of upper extremity arterial occlusion	Right brachial artery	Right brachial artery	Left axillary artery	Right brachial artery	Right ulnar artery	Right brachial artery	Right axillary artery
Symptoms of extremity occlusion	Pulseless, paleness, cold arm	Pulseless, paleness, cold arm	Pulseless, paleness, clod arm	Pulseless	Pulseless, paleness	Paresthesia, pain	Pulseless, cold arm
Etiology of stroke ^2^	Stroke of undetermined etiology	Cardio-embolism	Stroke of undetermined etiology	Cardio-embolism	Cardio-embolism	Cardio-embolism	Cardio-embolism

NIHSS: National Institutes of Health Stroke Score, mRS: modified Rankin Scale, ASPECTS: Alberta stroke program early CT score, i.v.: intravenous; CCA: common carotid artery, ICA: internal carotid artery, VA: vertebral artery. ^1^ Alteplase; before mechanical thrombectomy ^2^ According to the TOAST classification; assessed postinterventionally; ^3^ Patient #2 presented with several large vessel occlusions: occlusion of the left internal carotid artery, right common carotid artery and right vertebral artery; ^4^ Patient #3 presented with two large vessel occlusions: occlusion of the basilar artery and of the middle cerebral artery (M1-segment).

**Table 2:** Procedural characteristics.

**Characteristic**	Patient No.						
	1	2	3	4	5	6	7
Total procedure time [min]	85	71	83	81	68	60	87
Type of anesthesia	General anesthesia	Conscious sedation	Conscious sedation	General anesthesia	General anesthesia	General anesthesia	General anesthesia
	Craniocervical occlusion						
mTICI before treatment	0	0	0	0	2a	0	0
Total number of MT maneuvers	9	3	9	2	1	1	4
Number of MT maneuvers using SR	3	2	5	1	0	1	2
SRs used ^1^	Tiger XL, EmboTrap III 5 × 37, Eric 6 × 40	Trevo 4 × 20	EmboTrap II 4 × 20	pRESET 4 × 20	-	EmboTrap II 5 × 33	pRESET 6 × 30
Number of MT maneuvers using direct aspiration	6	1	4	1	1	0	2
mTICI after treatment	2b	2c (left), 3 (right)	3 (BA), 3 (MCA)	3	3	3	2b
Complications	No	No	No	No	No	No	Yes ^3^
Onset to groin puncture [min]	335	104	136	350 ^4^	NA	125	290
Procedure time [min]	102	82	105	51	59	31	228
	Upper extremity arterial occlusion						
TILI before treatment	0	0	0	0	0	0	0
Access devices ^2^	Merci 9F/Sofia 6F/Rebar 18/Traxcess 14	Merci 9F/Sofia 6F/Rebar 18/Traxcess 14	Merci 9F/Sofia 6F/Rebar 18/Traxcess 14	Neuron MAX 088/Sofia 6F /-/-	NeuronMax 088/ACE 68/3MAX/-	VISTA BRITE TIP 8F/Sofia 6F/Rebar 18/Traxcess 14	Guider Softip 8F/5MAX/Echelon 10, Trevo Pro 18/Traxcess 14
Total number of MT maneuvers	4	4	4	1	1	3	3
Number of MT maneuvers using SR	2	1	0	0	0	2	2
SRs used ^1^	Tiger XL, EmboTrap III 5 × 37, Eric 6 × 40	Neva-T	-	-	-	EmboTrap II 5 × 33	pRESET 6 × 30
Number of MT maneuvers using direct aspiration	2	3	4	3	1	1	1
TILI after treatment	2b	2b	2b	2b	0	3	2b
Complications	Yes ^5^	No	No	No	No	No	No
Procedure time [min]	80	41	24	21	13	42	50

MT: mechanical thrombectomy, SR: stent retriever, NA: Data not available ^1^ Guiding catheter/intermediate catheter/microcatheter/microwire; Merci, Guider Softip, Trevo Pro and Synchro (Stryker Neurovascular, Kalamazoo, MI, USA), Sofia and Traxcess (MicroVention, Aliso Viejo, CA, USA), Rebar and Echelon (Medtronic, Minneapolis, MN, USA), Neuron MAX, ACE, 3MAX and 5MAX ACE (Penumbra, Alameda, CA, USA) VISTA BRITE TIP (Cordis, Santa Clara, CA, USA); ^2^ Tiger XL (Rapid Medical, Yokneam, Israel), EmboTrap II/III (Cerenovus, Miami,FL, USA), pRESET (phenox, Bochum, Germany), Neva-T (Vesalio, Nashville, TN, USA); ^3^ Cervical dissection with only minor hemodynamic effect and good collateralization via the contralateral site, not requiring interventional treatment; ^4^ Wake-up stroke, last-seen well to groin puncture time is indicated; ^5^ Transient perforation of the distal brachial artery proximal to its bifurcation without any flow disruption or clinically visible or palpable hematoma.

**Table 3:** Outcome.

**Parameter**	Patient No.						
	1	2	3	4	5	6	7
Intracranial hemorrhage	No	No	No	No	No	No	Yes ^3^
NIHSS at discharge	22	10	15	4	20	0	10
Change of NIHSS ^1^	+13	−9	−9	−7	0	−12	−5
mRS at discharge	5	5	5	2	5	0	4
mRS at 3 months	4	5	Not available	2	6	0	5
Status of extremity on discharge ^2^	I	I	I	I	IIa	I	I

NIHSS: National Institutes of Health Stroke Score, mRS: modified Rankin Scale. ^1^ NIHSS at discharge minus baseline NIHSS (positive NIHSS indicates aggravation of symptoms, negative NIHSS indicates improvement in symptoms) ^2^ According to the Rutherford classification; ^3^ Hematoma occupying ≥ 30% of the infarcted tissue with occupying mass effect (PH2 according to the Heidelberg bleeding classification).

## Data Availability

All relevant data are included in this article.
